# Photoacoustic imaging with low-cost sources; A review

**DOI:** 10.1016/j.pacs.2019.01.004

**Published:** 2019-02-19

**Authors:** Mohsen Erfanzadeh, Quing Zhu

**Affiliations:** a Department of Biomedical Engineering, University of Connecticut, Storrs, CT 06269, USA; b Department of Biomedical Engineering, Washington University in St. Louis, St. Louis, MO 63130, USA; c Department of Radiology, Washington University School of Medicine, St. Louis, MO 63110, USA

**Keywords:** Photoacoustic imaging, Low-cost, Laser diode, Light emitting diode, Medical imaging

## Abstract

Benefitting from advantages of optical and ultrasound imaging, photoacoustic imaging (PAI) has demonstrated potentials in a wide range of medical applications. In order to facilitate clinical applications of PAI and encourage its application in low-resource settings, research on low-cost photoacoustic imaging with inexpensive optical sources has gained attention. Here, we review the advances made in photoacoustic imaging with low-cost sources.

## 1 Introduction

As a hybrid imaging modality, photoacoustic systems detect ultrasound signals that are thermoelastically induced by absorption of time-varying optical energy [1]. Upon absorption of pulsed or modulated optical energy, tissue undergoes a time-varying thermal expansion-relaxation process, which leads to the generation of acoustic waves in the tissue [[1], [2], [3], [4]]. Therefore, photoacoustic imaging (PAI) benefits from advantages of wavelength-dependent absorption selectivity in optical excitation and acoustic resolution of ultrasound detection.

Similar to ultrasound imaging, axial resolution in PAI mainly depends on the ultrasound transducer frequency and bandwidth. Absorption of short pulses of light (nanosecond range) generates broadband photoacoustic (PA) signals, providing frequency contents as high as several hundreds of megahertz [3]. Transducers with high frequencies (approaching or even exceeding 100 MHz [5]) and bandwidth are capable of providing higher axial resolutions (few tens of μm). On the other hand, high frequency signals suffer from higher acoustic attenuation resulting in limited imaging depth. In contrast, transducers with lower frequencies detect the low frequency PA signals, which are stronger than that of high frequency, with lower axial resolutions (tens or few hundreds of μm) but suffer less acoustic attenuation and can detect signals from deep-lying absorbers. Generally, photoacoustic tomography (PAT) that aims to reconstruct deeper targets utilizes lower frequency transducers and photoacoustic microscopy (PAM) that aims to image shallower targets utilizes higher frequency transducers. It shall also be noted that sufficient light energy should reach the deep-lying targets in order to generate PA signals and the imaging depth in PAI may be limited by either penetration depth of excitation light and/or detection sensitivity of the ultrasound transducer. Moreover, if the penetration depth is smaller than the axial resolution of the transducer, axial resolution may not apply to the system.

The lateral resolution in PAI systems depends on the focusing of excitation (light) or detection (ultrasound). Optical resolution photoacoustic microscopy (OR-PAM) focuses the excitation light on the tissue, therefore its lateral resolution is governed by the light wavelength, numerical aperture of the focusing mechanism, and limitations of optical focusing such as aberrations [1]. OR-PAM systems usually have the highest lateral resolutions among PAI systems but have lower depth of penetration mostly because of tissue scattering and lower light power used to avoid tissue damage. In acoustic resolution photoacoustic microscopy (AR-PAM), light is either unfocused or weakly focused, instead focused ultrasound transducers are utilized. In other words, in AR-PAM lateral resolution is governed by the focal spot of ultrasound transducer. Increasing the numerical aperture and center frequency of the ultrasound transducer increases the lateral resolution of AR-PAM systems while compromising the imaging depth. Although OR-PAM systems may also use focused ultrasound detection for enhanced sensitivity, their lateral resolution is governed by the optical focusing [1,6]. AR-PAM systems usually have lower lateral resolutions compared to OR-PAM but higher depth of penetration. Photoacoustic microscopy systems use raster scanning [1,7] while in photoacoustic tomography electronic steering of ultrasound arrays or rotating a single ultrasound transducer around the sample combined with tomography algorithms are used for image reconstruction. Lateral resolution of PAT systems is lower than PAM systems as PAT aims to image a large volume of the sample and probe deeper targets.

With excitation sensitivity to optical absorption, PAI systems using visible or near infrared (NIR) light are capable of resolving blood vasculature given the higher absorption of hemoglobin compared to other tissue constituents in these wavelength ranges [8]. Therefore, PAI can be used for detection of angiogenesis, which is the irregular growth of blood vasculature in the vicinity of tumors [9,10]. This capability has led to numerous studies demonstrating the potentials of PAI for monitoring and diagnosis of various cancer types including ovarian, breast, skin, colorectal, pancreatic, thyroid, prostate, and cervical cancers [[11], [12], [13], [14], [15], [16], [17], [18], [19], [20], [21]].

Because PA signals generated from absorption of pulsed light are inherently broadband, PAI can provide high axial resolution allowing for creating three-dimensional high-resolution images. Therefore, the most common light sources in PAI are pulsed lasers, usually with nanosecond pulse durations for improved PA signal generation efficiency and preserving the axial resolution governed by the transducer specifications [1,3]. However, such lasers are generally large and relatively expensive. In order to further facilitate clinical utility of PAI and also encourage its application in low-resource settings, it is beneficial to develop PAI systems with small and inexpensive light sources. To this end, many researchers have developed PAT, OR-PAM, and AR-PAM systems utilizing low-cost sources and different laser manufacturing companies have focused on development of suitable low-cost light sources for PAI [[22], [23], [24], [25], [26]]. Developing low-cost imaging systems for improving clinical applications and applications in low-resource settings is a topic of interest in other optical imaging modalities as well [[27], [28], [29], [30], [31], [32], [33], [34]]. Here, we review the advances in development and applications of inexpensive PAI systems in recent years. The paper will be divided into three main parts of OR-PAM, AR-PAM, and PAT systems with low-cost sources with subsections based on the light sources used in the system.

## 2 Optical resolution photoacoustic microscopy systems with low-cost sources

A large number of low-cost PAI systems reported to date are in OR-PAM configuration. In fact, inexpensive pulsed laser diodes (LD) or light emitting diodes (LED) have limited energy compared to conventional pulsed solid-state lasers and using them in OR-PAM setting which requires the least excitation energy amongst PAI systems is a straightforward choice. Of course, this limitation in energy is also accompanied with large divergence angles of laser diodes and LEDs and challenges the low-loss focusing of the beam for optical resolution photoacoustic microscopy [35]. In this section, OR-PAM systems with low-cost sources are summarized. These systems can also be subcategorized into visible and NIR low-cost OR-PAM systems.

### 2.1 Visible low-cost OR-PAM systems

Visible OR-PAM systems benefit from high absorption of blood in the blue (˜400 nm) region. This high absorption, to some extent, compensates the low available energy of visible laser diodes and LEDs. However, it should be mentioned that shorter wavelengths are more susceptible to light scattering and tissue chromophores have high absorption in the blue region, which limits the penetration depth compared to NIR wavelengths.

**Fig. 1:**
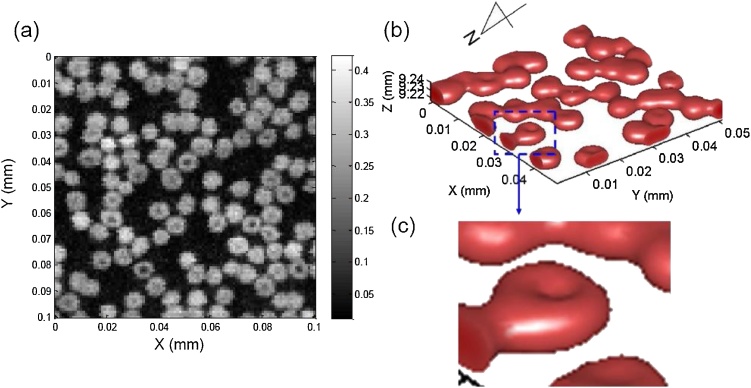
(a) Two-dimensional maximum amplitude projection PAM image of red blood cells over a 100 μm × 100 μm area. (b) 3D photoacoustic image of red blood cells over a 50 μm × 50 μm × 20 μm volume. (c) Zoomed in from (b) showing the biconcave structure of red blood cells. Reprinted from Ref. [36].

**Fig. 2:**
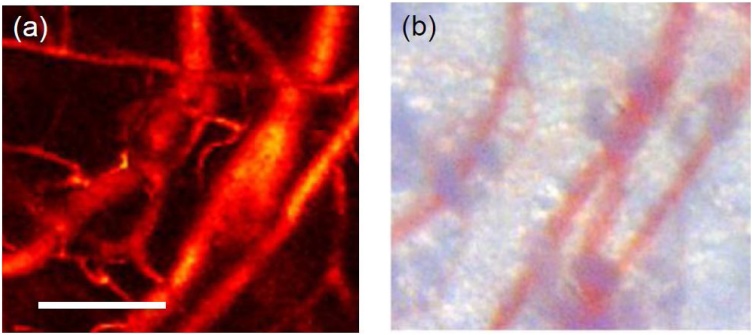
(a) PAM image of a mouse ear, scale bar is 200 μm. (b) 200x optical microscopic image of the sample. Reprinted from Ref. [36].

In 2014, Li et al. [36] developed a visible laser diode based OR-PAM system using the 405 nm laser diode from a commercial Blue-ray DVD player (iHBS212, Liteon, Taiwan). The laser was driven to have 30 KHz repetition rate and tunable pulse width of 10–30 ns. Images were formed using raster scanning with a precise motorized stage. Images of red blood cells (Fig. 1) and mouse ear microvasculature (Fig. 2) demonstrated the imaging capability of the system. 1500 averaging per A-line was performed for resolving red blood cells and 3000 averaging was required for resolving the microvasculature on the mouse ear.

**Fig. 3:**
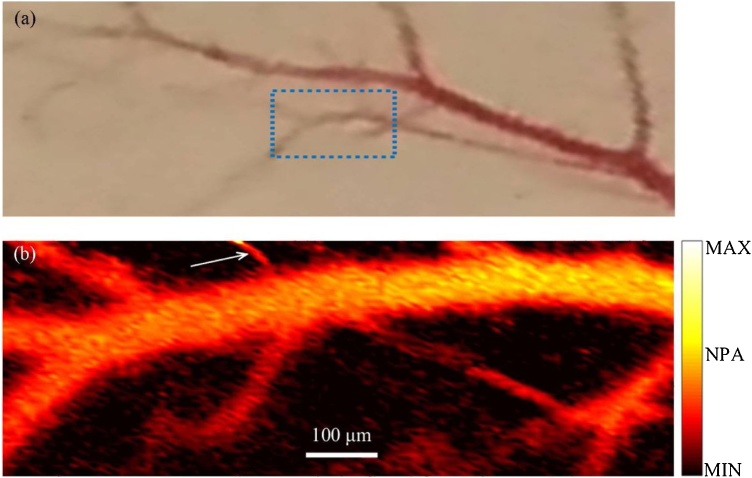
Photograph (a) and PAM image of ex vivo subcutaneous microvasculature on a mouse back. Reprinted from Ref. [37].

In 2015, using a 405 nm laser diode (Sony, Japan) Zeng et al. [37] developed a visible laser diode OR-PAM system. The laser diode was pulsed with 1 KHz repetition rate and carried ˜52 nJ of energy in pulses with 174 ns pulse width. An aspheric objective lens system provided a lateral resolution of ˜0.95 μm. Images were formed by raster scanning using a motorized stage and 512 averaging were performed when imaging biological tissue. PAM images of *ex vivo* subcutaneous microvasculature on a mouse back were acquired (Fig. 3), which showed the potential of this system for superficial imaging of blood vasculature. Both reported visible laser diode based OR-PAM systems were able to provide high resolution images but suffered from shallow depth and low excitation energy (requiring hundreds of averaging).

### 2.2 NIR low-cost OR-PAM systems

Compared to visible light, blood has lower absorption in NIR but still higher than other tissue constituents. On the other hand, NIR light shows higher penetration depth given the lower scattering and absorption compared to the visible range. Moreover, available NIR pulsed laser diodes have much higher power compared to visible (especially blue) pulsed laser diodes due to manufacturing constraints, a fact that partially compensates the lower absorption coefficient of hemoglobin in NIR region compared to the visible light. On the other hand, in order to provide higher energy and also allow for short pulses (10 s of ns), high power NIR PLDs are manufactured as multiple stacked emitters leading to a rather large emitting area with a beam comprised of multiple bars physically separated from one another at the source. This physical separation further challenges the low-loss collimation and tight focusing of the beam for PAM applications. As a result, initial reports of such PAM systems had low single-shot signal to noise ratio (SNR) and required multiple averaging resulting in low imaging speed, while more recent investigations have shown improved light delivery for faster PAM imaging. It shall be noted that given the increase in emitting area with the increase in energy, highest power NIR PLDs that are used in PAT systems will basically suffer more energy loss during collimation and focusing, hence their application in OR-PAM is not common.

In 2012, Zeng et al. [38] used a pulsed laser diode at 905 nm with light focused using a collimating and focusing lens and provided a lateral resolution of about 500 μm. Although this lateral resolution is much weaker compared to standard OR-PAM systems, because the mechanism governing the lateral resolution is the optical focusing, the system is categorized here as OR-PAM here. The ultrasound transducer is a 1.95 MHz transducer with a focal length of 37.5 mm and 10 mm active element diameter that results in a focal spot of about 2.9 mm, hence weaker than the optical focusing. The laser was pulsed at 0.8 KHz repetition rate and 100 ns pulses carried 5.6 μJ of energy. Images of vessels filled with black ink embedded in tissue-mimicking phantoms showed the initial capability of PAI imaging with the low-cost NIR pulsed laser source. Given the NIR wavelength, low transducer frequency, and weak optical focusing, the system is capable of resolving ink-filled vessels with more than 15 mm depth when each A-line is averaged 256 times.

In 2013, Zeng et al. [39] developed an OR-PAM system using a 905 nm pulsed laser diode with 0.8 KHz repetition rate, 100 ns pulse width, and 4.9 μJ of energy per pulse with an improved light focusing scheme that resulted in a lateral resolution of about 1.5 μm, well fit in the OR-PAM regime. In this focusing scheme, the laser diode light passed a 4-mm focal length aspheric objective lens after it was weakly focused by a collimation-focusing set of lenses with a long 15 mm focal length. An unfocused 4.53 MHz, 152.8% bandwidth ultrasound transducer provided an axial resolution of approximately ˜96 μm for the system and 128 times averaging was performed for each A-line. Moreover, the complete imaging system was assembled in a portable and compact equipment. PAM Images of 4 μm carbon fibers demonstrated the imaging capability and resolution of this system.

In 2014, Wang et al. [40] demonstrated imaging biological tissue with a NIR low-cost OR-PAM system. The OR-PAM system utilized a 905 nm pulsed laser diode with 1 KHz repetition rate and 124 ns pulse width providing ˜3 μJ energy per pulse to the sample. Light focusing was performed by a 60x microscope objective lens (0.7 numerical aperture (NA)) after a collimating tube and provided 7 μm lateral resolution. A 3.5 MHz unfocused ultrasound transducer detected the ultrasound signals and each A-line was averaged 128 times. PAM images of polyethylene tubes filled with whole blood and vasculature on an *ex vivo* mouse ear demonstrated the capability of the low-cost NIR OR-PAM system for imaging biological tissue.

**Fig. 4:**
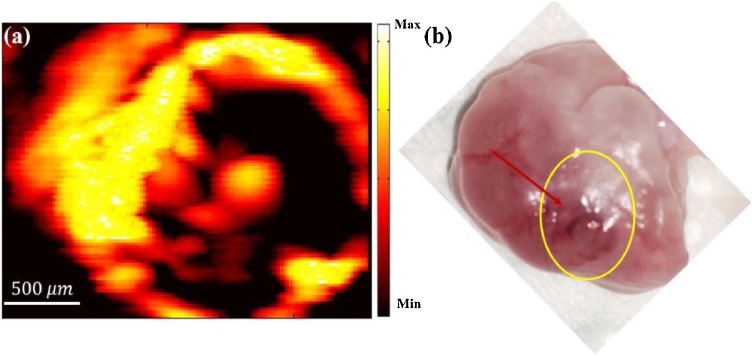
PAM image (a) and photograph (b) of a porcine ovarian tissue. Reprinted from Ref. [41].

In 2016, Erfanzadeh et al. [41] suggested an optical scheme to efficiently collimate and focus the beam of a 905 nm high power pulsed laser diode comprised of multiple separated active areas. Here, light collimation was performed by an aspheric lens followed by two cylindrical lenses, each collimating the beam in one direction perpendicular to the other. The light remained collimated for about 400 mm until it reached an aspheric objective lens with 0.71 NA. The system had a lateral resolution of ˜40 μm A 3.5 MHz unfocused ultrasound transducer was used to detect the photoacoustic signals and 128 times averaging was performed on each A-line. PAM images of polyethylene tubes filled with whole blood showed ˜20 dB improvement in SNR compared to images of similar targets reported by Wang et al. [40,41]. Moreover, images of vasculature on *ex vivo* porcine ovarian tissue (Fig. 4) with 25 dB SNR, demonstrated the potentials of using low-cost OR-PAM systems for imaging and characterization of ovarian cancer.

In 2017, Hariri et al. [42] used a combination of an achromatic convex lens for light collection, a microscope objective for spherically shaping the beam, and a second achromatic convex lens to focus the beam of a low-energy (6 W maximum peak power) 905 nm pulsed laser diode for PAM imaging. This study showed PAM imaging in both transmission mode (incident light and detector at 180 degrees with respect to one another) and reflection mode (incident light and detector on the same side of the target, at 40 degrees with respect to one another in this setup), which is a preferred configuration. A lateral resolution of approximately 100 μm was reported for either configuration. The laser was pulsed at 10 KHz with 55 ns pulse width. An unfocused 10 MHz ultrasound transducer detected the ultrasound signals and 5000 averaging was performed for imaging mouse skin vasculature *ex vivo* with 2 dB SNR in the reflection mode. An adaptive denoising filtering approach has also been reported to enhance the SNR of PA signals obtained from low energy pulsed laser diodes [43].

**Fig. 5:**
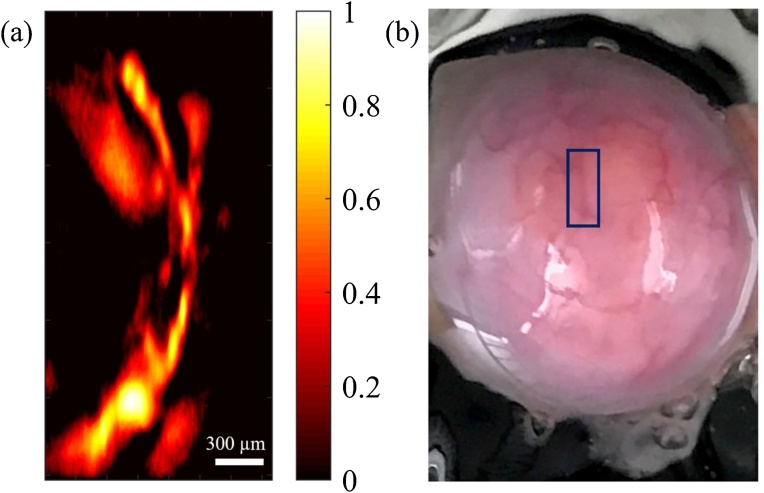
PAM image (a) and photograph (b) of porcine ovarian tissue obtained by the laser scanning laser diode based OR-PAM system. Color bar represents normalized photoacoustic signal. Reprinted from Ref. [44].

The aforementioned low-cost OR-PAM systems (both visible and NIR) utilized motor scanning of the sample and also performed multiple averaging in data acquisition. Erfanzadeh et al. [44,45] reported a laser scanning laser diode based OR-PAM system with no signal averaging for imaging biological tissue. The 905 nm laser light was pulsed at 1 KHz repetition rate with 50 ns pulse width. An aspheric lens and two pairs of perpendicular cylindrical beam expanders provided suitable collimation and small enough beam spot to allow for two dimensional galvo-scanning of the beam in a 1-inch aspheric focusing lens and provided the lateral resolution of ˜ 21 μm. Light energy from the laser was measured ˜ 16 μJ and about 13 μJ was delivered to the tissue. An unfocused 3.5 MHz ultrasound transducer detected the ultrasound signal and no averaging was performed in data acquisition, leading to approximately 370 A-lines per second. Nevertheless, the same laser diode can be driven with repetition rates as high as 20 KHz with 50 ns pulse width and can potentially provide further improved imaging speeds. The depth of penetration in biological tissues was measured to be ˜2 mm. PAM images of *ex vivo* mouse ear with ˜12 dB SNR and porcine ovary with ˜18 dB SNR (Fig. 5) were demonstrated, showing the capability of the system for imaging biological samples including ovarian tissue, providing the potential for imaging and characterization of ovarian cancer with the low-cost and fast laser scanning OR-PAM system.

## 3 Acoustic resolution photoacoustic microscopy systems with low-cost sources

Although less frequently compared to OR-PAM systems, PAM systems relying on acoustic resolution using low-cost sources have also been reported. In 2017, Dai et al. [46] overdrove a 405 nm LED to provide 1.2 μJ energy in 200 ns pulses with 40 KHz repetition rate. Light was weakly focused on a 1 mm^2^ area and a focused ultrasound transducer with a center frequency of 2.25 MHz and focal length of 25.4 mm resulted in a lateral resolution of ˜150 μm. While the sample was fixed, the light beam and the ultrasound transducer were scanned with a linear stage to form images. Images of vasculature in a mouse ear *in vivo* were acquired with 4000 times averaging (resulting in ˜ 10 A-lines per second) and ˜ 14 dB SNR.

**Fig. 6:**
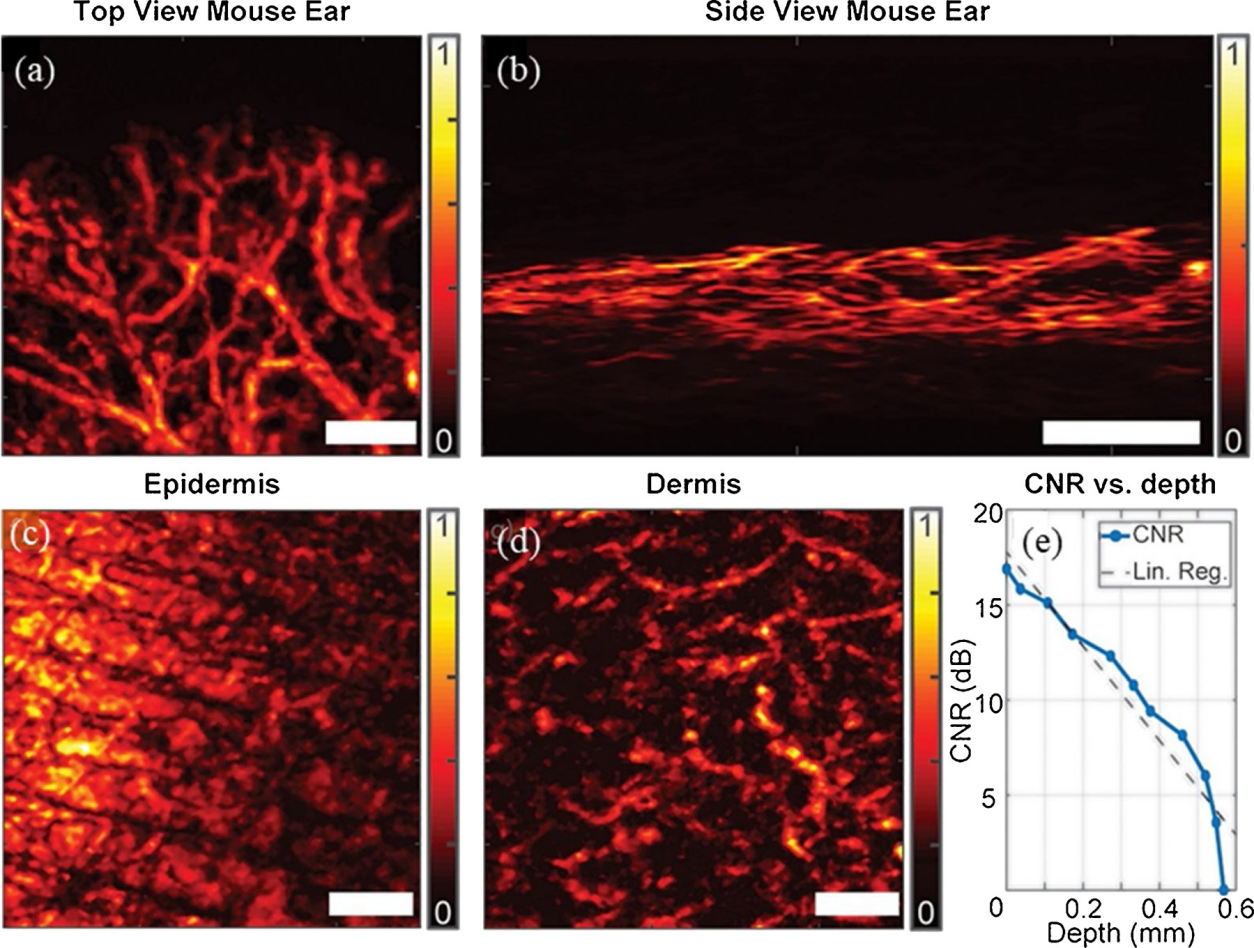
Top view (a) and side view (b) AR-PAM images of a mouse ear *in vivo* and PAM images of a human forearm *in vivo* at (c) epidermis (0–280 μm) and (d) dermis (280–550 μm) depth. (e) Contrast to noise ratio in human forearm as a function of depth. Scale bars represent 1 mm. Reprinted from Ref. [47].

In 2018, Stylogiannis et al. [47] overdrove continuous wave (CW) lasers at different wavelengths, ranging from 445 nm to 830 nm, to provide 10 ns pulses carrying ˜ 200 nJ energy per pulse with as high as 625 KHz repetition rate. The laser light was weakly focused to an elliptical shape with 880 μm and 720 μm axes (72 nJ energy per pulse after the optical elements) and a focused transducer with 28.8 MHz center frequency, 112% bandwidth, and f-number of 1.07 provided a lateral resolution of 110 μm and an axial resolution of 33 μm. The light delivery optical components and the transducer were fixed on a probe, which was two-dimensionally scanned by a motorized stage to form images while the sample was fixed at place. The ultrasound transducer and optical elements are tilted at 60 degrees, therefore the ultrasound signals are detected in the reflection mode configuration. PAM images of vasculature in a mouse ear *in vivo* and a human forearm *in vivo* at epidermis and dermis levels were acquired using the 450 nm laser. Mouse ear imaging was performed with 625 KHz repetition rate and human forearm imaging was performed at 156 KHz repetition rate and both utilized 500 times averaging (Fig. 6). Data acquisition for the mouse ear imaging with 625 KHz repetition rate lasted approximately 97 s (˜410 A-lines per second).

## 4 Photoacoustic tomography systems with low-cost sources

Photoacoustic tomography systems employing low-cost light sources such as laser diodes, Xenon flash lamps, and LEDs have been reported in the literature.

### 4.1 PAT with pulsed laser diodes

State-of-the-art highest power NIR PLDs can generate up to 4 mJ energy per pulse with 100 ns pulse width (also configurable to as low as 30 ns pulse width and 1 mJ energy per pulse). Although this energy is lower than the energy of pulsed solid-state lasers when used in PAT (˜20 mJ), the higher repetition rate of PLDs (in the KHz range) allows for averaging and increasing the SNR [48].

**Fig. 7:**
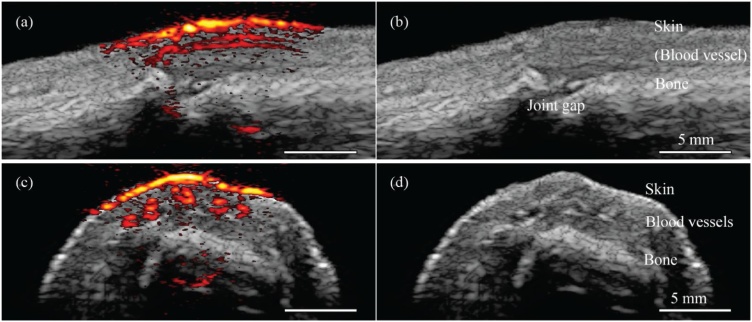
Co-registered photoacoustic and ultrasound images of a human proximal interphalangeal joint in sagittal (a) and transverse (c) view. (b) and (d) are anatomical-only ultrasound images from (a) and (c), respectively. Reprinted from Ref. [51].

Initial studies of assessing laser diodes in PAT systems for imaging phantoms and blood vessels were reported as early as 2006 [49,50]. In 2014, Daoudi et al. [51] developed a handheld probe for co-registered ultrasound and photoacoustic tomography using an 805 nm laser diode with 10 KHz repetition rate, 130 ns pulse width, and 0.56 mJ energy per pulse. Generation and detection of pulse-echo ultrasound signals and detection of photoacoustic signals were performed by a 7.5 MHz linear array ultrasound transducer with 100% -6 dB bandwidth. *In vivo* images of human proximal interphalangeal (PIP) joint were presented to demonstrate the imaging capability of the system (Fig. 7). Using this handheld probe, Arabul et al. [52] demonstrated the application of photoacoustic imaging in detecting intraplaque hemorrhage in carotid artery lesions. This *ex vivo* study revealed the advantage of PAI compared to plane wave ultrasound (PUS) imaging in detecting intraplaque hemorrhage suggesting the potential for future *in vivo* applications.

**Fig. 8:**
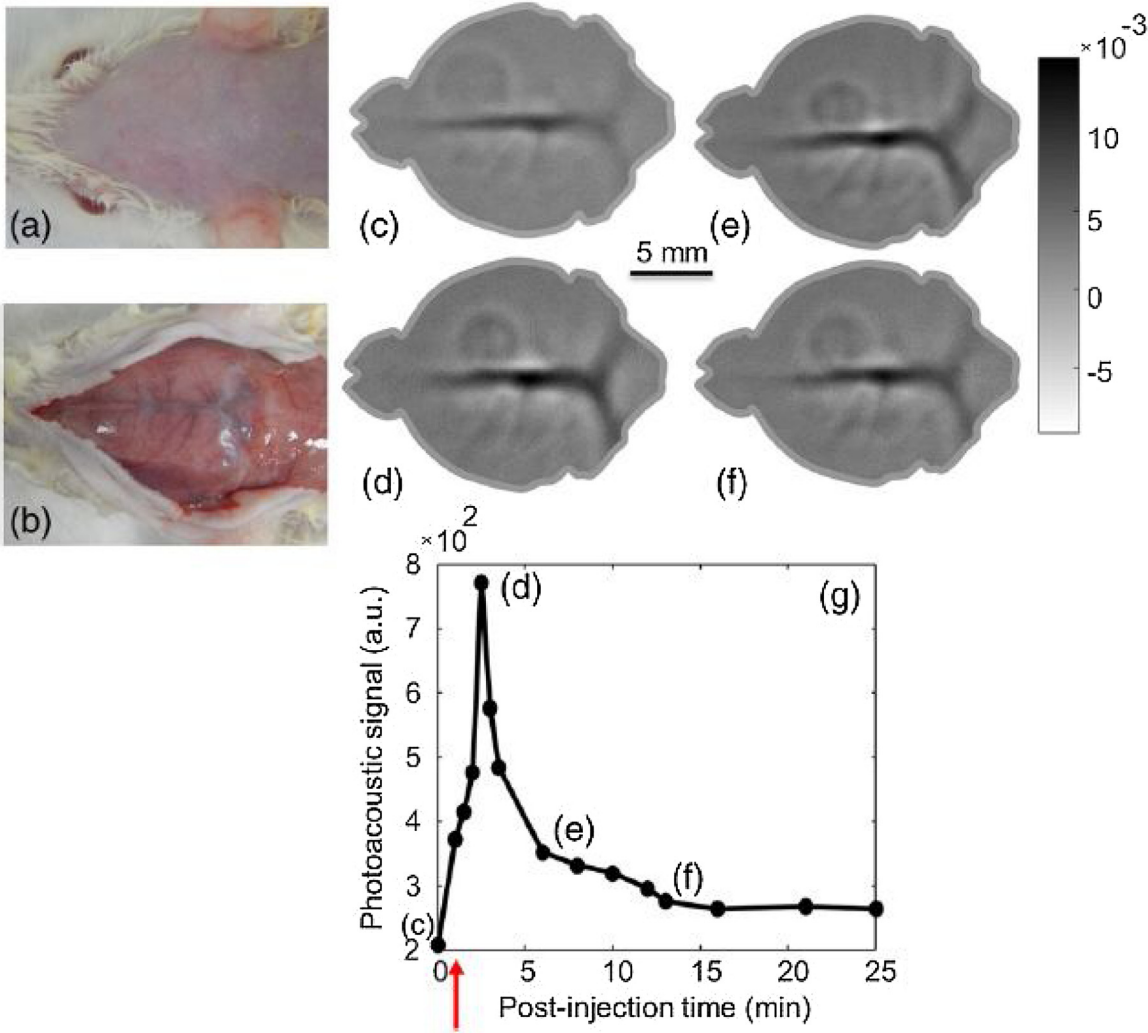
Photograph of rat brain, (a) before and (b) after removal of the scalp. (a–d) PAT images of rat brain 0, 2, 6, and 13 min after injection of ICG, respectively. (g) Photoacoustic signal level from ICG at the superior SS over time. Reprinted from Ref. [55].

In 2015, Upputuri and Pramanik [53,54] reported a portable and low-cost PAT system with a pulsed laser diode at 803 nm, 7 KHz repetition rate, and 136 ns pulse width carrying 1.4 mJ energy per pulse. In this system, a single element ultrasound transducer (either 2.25 MHz or 5 MHz) rotated around the sample and collected 360-degree data and this data set was used to reconstruct PAT images using delay-and-sum back projection reconstruction algorithms. The initial report demonstrated the capability of imaging tubes filled with blood and indocyanine green (ICG) as deep as 3 cm below chicken breast tissue with ˜ 10 dB SNR [53], followed by a study comparing the performance of two similar systems; one with a pulsed laser diode source and the other with a conventional optical parametric oscillator (OPO) pulsed laser source [54]. The similarity of phantom results indicated the feasibility of using a pulsed laser diode as a low-cost excitation source for PAT, especially given the possibility of multiple averaging due to the significantly higher repetition rate of the laser diode compared to the OPO. In 2017, Upputuri et al. [55,56] reported imaging rat brain *in vivo* and monitoring the uptake and clearance of injected ICG (Fig. 8) using a similar system and image acquisition times as short as 5 s. The results clearly indicate the capability of this low-cost and portable PAT system for monitoring the uptake of ICG in cortex vasculature of small animals revealing its potential for pre-clinical applications. Moreover, Kalva et al. [57] have presented a second generation of this PLD-based PAT system using eight single element transducers rotating around the sample, hence reducing the image acquisition time to 0.5 s compared to 5 s in the first generation without compromising image quality.

### 4.2 PAT with a Xenon flash lamp

In 2016, Wong et al. [58] reported using a Xenon flash lamp as the excitation source for PAT. The repetition rate of the Xenon lamp could be controlled between 10–100 Hz and 3 mJ of energy was carried in pulses with 1 μs pulse width. A focused single ultrasound transducer revolving around the target and a ring-shaped ultrasonic transducer array were evaluated for detection of ultrasound signals and the ring-shaped transducer at 0.5 MHz center frequency was used for imaging blood filled tubing 1 cm deep under a tissue mimicking phantom (1000 times averaging) and a mouse body *in vivo* (5000 times averaging) to improve SNR with more averaging and avoid mechanical scanning.

### 4.3 PAT with light emitting diodes

In 2016, Allen and Beard [59] demonstrated the potential of safely overdriving LEDs up to 20 times higher than their current limit and reaching up to 9 μJ of energy in 200 ns pulses of a 623 nm LED with 500 Hz repetition rate (0.01% duty cycle to facilitate safe overdriving) and images of blood-filled tubes immersed in an Intralipid solution (5000 times averaging) were presented to show the imaging capability of the system. The authors also demonstrated using a single multi-wavelength LED (460 nm, 530 nm, 590 nm, and 620 nm). Moreover, they demonstrated the improvement in SNR and simultaneous acquisition at different wavelengths utilizing a coded excitation scheme taking benefit of the flexibility in controlling LEDs compared to conventional lasers.

Studies utilizing a commercially available LED based photoacoustic tomography systems from PreXion Corporation (Tokyo, Japan) for different applications have been reported recently. These systems utilize multiple LED arrays at different wavelengths with variable repetition rates and pulse widths located on both sides of a linear array ultrasound transducer to form photoacoustic imaging and can work in co-registered ultrasound/photoacoustic mode or at a higher speed in photoacoustic-only mode for dynamic photoacoustic studies [60].

In 2018, Hariri et al. [61] utilized a system with LED arrays at two wavelengths (690 nm and 850 nm) and carefully characterized its performance for biomedical imaging. They then evaluated detection of photoacoustic contrast agents and imaging of the vasculature in a rabbit eye *ex vivo* using the system. The feasibility of using such system for imaging human placental vasculature was also investigate by Maneas et al. [62]. Moreover, Mozaffarzadeh et al. [63] evaluated a double-stage delay-multiply-and-sum image reconstruction method in order to improve the quality of images obtained by array scanner LED-based photoacoustic systems, yielding enhanced lateral and temporal resolution, contrast ratio, and depth of penetration compared to images reconstructed using delay-and-sum and delay-multiple-and-sum reconstruction methods.

**Fig. 9:**
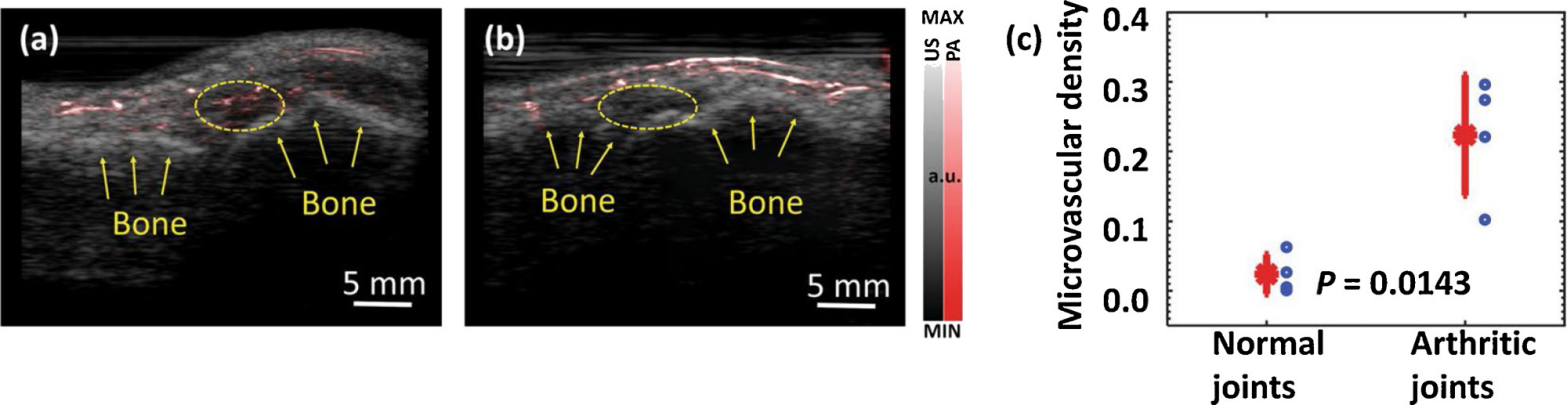
Co-registered photoacoustic and ultrasound images an arthritic (a) and healthy (b) human MCP joint. (c) Comparison of microvasculature density at normal and arthritic joints. Reprinted from Ref. [64].

**Fig. 10:**
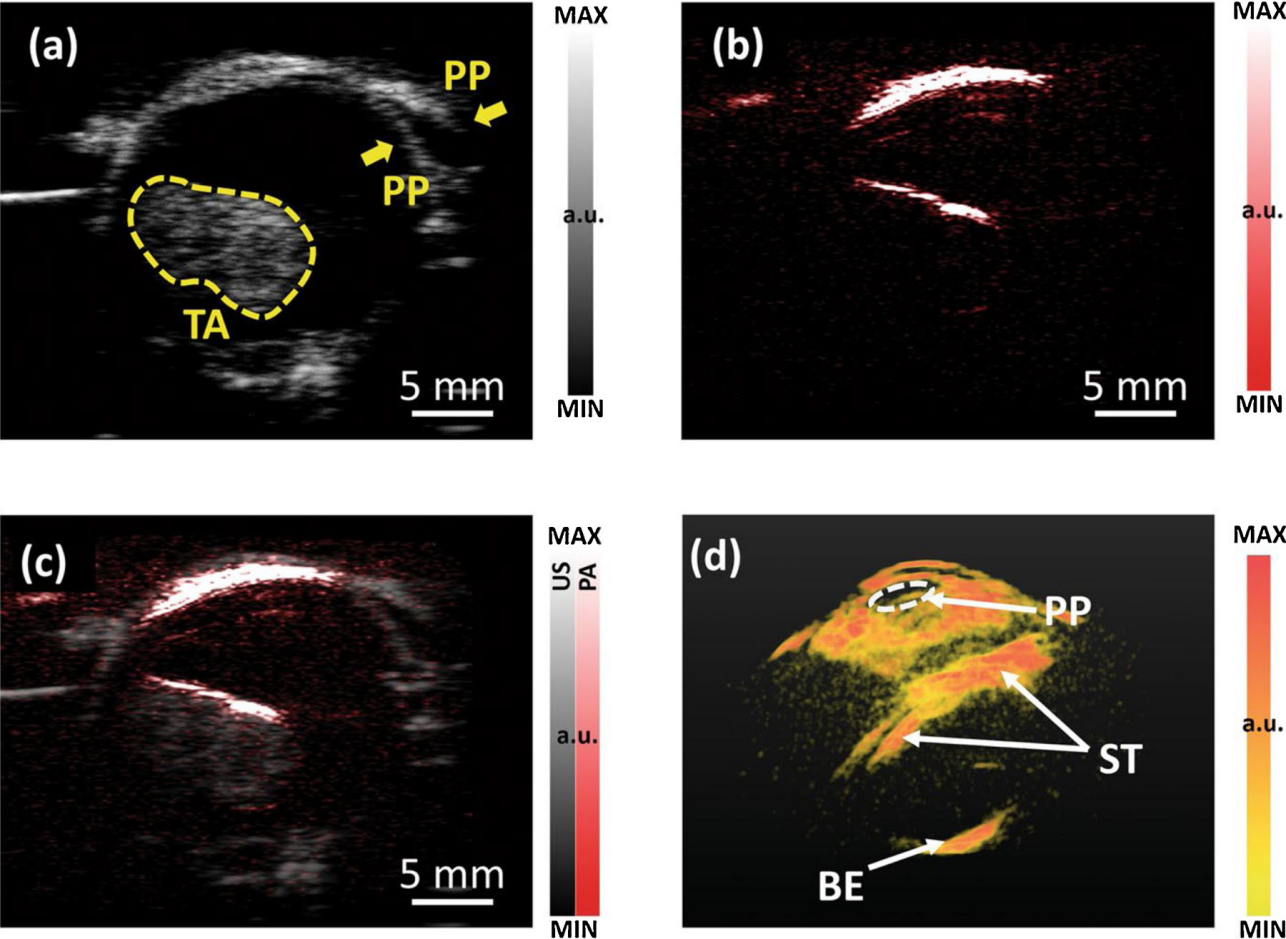
Ultrasound (a), photoacoustic (b), and co-registered (c) B-scan of an ex vivo human ocular globe with a choroidal melanoma tumor. (d) Perspective view of a 3D photoacoustic image of the sample. Parts identified on the images are pupil (PP), tumor area (TA), surface of the tumor (ST), and back of the eye (BE). Reprinted from Ref. [64].

In 2018, Zhu et al. [64] utilized an upgraded version of similar system with 4 different wavelengths (470 nm, 620 nm, 690 nm, and 850 nm) and demonstrated its applications in imaging the vasculature and oxygen saturation in human finger *in vivo*, human foot *in vivo*, differentiating healthy human metacarpophalangeal (MCP) joint and those affected by arthritis *in vivo* (Fig. 9), and human ocular globe with a choroidal melanoma tumor *ex vivo* (Fig. 10). These results reveal the potentials of the LED based photoacoustic tomography system for further clinical applications.

## 5 Conclusions

In this review article, we have provided an overview of PAI systems utilizing low-cost pulsed sources as alternatives for expensive and bulky pulsed lasers. A summary of PAI systems utilizing low-cost pulsed sources categorized based on the imaging modalities (OR-PAM, AR-PAM, and PAT) and the light sources is provided in Table 1.

**Table 1:** Summary of PAI systems using pulsed low-cost sources.

Modality	Sources	Advantages	Disadvantages	Biological samples
	Visible laser diode [36,37]	High lateral resolution	Limited penetration depth	Red blood cells
			Low imaging speed	Mouse ear *ex vivo*
OR-PAM	NIR laser diode [38,39,40,41,42,43,44,45]	High power PLDs, single shot acquisition possible	Challenging low-loss focusing	Mouse ear *ex vivo*
		Shown in laser scanning mode with no mechanical scanning and no averaging	Challenging to efficiently implement reflection mode configuration	Mouse skin *ex vivo*
				Porcine ovary *ex vivo*
	Overdriven visible LED [46]	Miniature LED	Low lateral resolution	Mouse ear *in vivo*
		Relatively high repetition rate	Long pulses	
			Limited power	
AR-PAM	Overdriven CW laser diode [47]	High repetition rate	Requires mechanical scanning	Mouse ear *in vivo*
				Human forearm *in vivo*
		Multi-wavelength		
		Short pulses		
		Potential for high imaging		
		speed		
		Inherently compatible with reflection mode		
	NIR laser diode [49,50,51,52,53,54,55,56]	Handheld probe	Limited penetration depth	Human PIP joint *in vivo*
				Intraplaque hemorrhage in carotid artery lesions *ex vivo*
		Co-registered PA/ultrasound		
				Rat brain *in vivo*
	Xenon flash lamp [58]	Simple setup	Long pulses	Mouse body *in vivo*
PAT	LED [59,60,61,62,64]	Multi-wavelength	Limited penetration depth	Rabbit eye *ex vivo*
				Human placental vasculature *ex vivo*
		Short pulses		
				Human finger *in vivo*
				Human foot *in vivo*
				Human arthritic MCP joint *in vivo*
		Handheld probe		
				Human ocular globe *ex vivo*
		Co-registered PA/ultrasound		

It is worth noting that frequency domain PAI systems using continuous-wave modulated laser diodes and LEDs have also been developed for biological and industrial applications [2,4,[65], [66], [67], [68], [69], [70], [71], [72], [73]]. In this review, however, we have focused on PAI systems with pulsed low-cost sources. A comparison between performance of PAI systems with pulsed and modulated sources can be found in Ref. [74].

PAI systems with low-cost pulsed sources have been designed in ORPAM, ARPAM, and PAT configurations with various wavelengths, resolutions, and imaging depths and have shown potentials for clinical and pre-clinical applications. For microscopic applications, in order to facilitate *in vivo* clinical applications, reflection mode PAM systems with high resolution and real time image acquisition are yet to be developed, an achievement that requires further investigations into improved light delivery methods, beam shaping [75], and scanning techniques. Tomography systems would also benefit from improved SNR, depth of penetration, and imaging speed as well as further clinical evaluations more thoroughly investigating the potentials of low-cost PAT systems for disease diagnosis and monitoring. Contrast agents with strong absorption in the NIR wavelength range and suitable for *in vivo* imaging can also benefit clinical applications of low-cost PAI system [76], [77], [78], [79], [80].

## Conflict of interest

None.
